# Study on the damage characteristics of gas-bearing shale under different unloading stress paths

**DOI:** 10.1371/journal.pone.0224654

**Published:** 2019-11-06

**Authors:** Yintong Guo, Lei Wang, Xin Chang

**Affiliations:** State Key Laboratory of Geomechanics and Geotechnical Engineering, Institute of Rock and Soil Mechanics, Chinese Academy of Sciences, Wuhan, China; China University of Mining and Technology, CHINA

## Abstract

In order to understand the influence of unloading on the mechanical properties of shale rock, triaxial unloading tests under different stress paths were conducted. In this paper, three types of tests are completed, including: 1) Conventional triaxial compression test;2) Pre-peak constant maximum principal stress-unloading confining pressure test with different initial confining pressures and rates;3) Increasing axial stress-unloading confining pressure test. The deformation and rupture modes characteristics of shale sample under different unloading stress paths were obtained. Research results show that: 1) The confining pressure effect is obvious and the peak strength increases with the increase of initial confining pressure, under conventional triaxial compression test, the samples show obvious elastic-plastic characteristics; Under unloading confining pressure test, it shows obvious elastic brittleness characteristics.2) Compared with conventional triaxial compression test, unloading confining pressure is more prone to deformation and rupture, and the damage is more serious. Under same initial stress level, the brittle characteristics in unloading confining pressure are more obvious and the expansion is more intense. 3) Under same unloading stress path, the higher the initial confining pressure is, the more severe the sample failure is. With the increase of unloading rate, the rupture degree of the sample becomes more complex.4) The brittle rupture characteristic increases with the increase of unloading rate and initial confining pressure. Increasing axial stress-unloading confining pressure, various types of tensile and shear fractures with different mechanisms are well developed. These conclusions reveal loading and unloading mechanical properties of gas-bearing shale under different stress paths; it provides theoretical basis for horizontal drilling, fracturing design and long-term fracturing effect analysis of shale gas reservoirs.

## 1. Introduction

With the rapid development of national economic construction, the demand for clean energy is increasing [1–2]; shale gas resources have become one of the important energy sources. From the mechanical mechanism analysis, the unloading behavior is the main factor in deep oil and gas drilling engineering, and the mechanical properties of rock mass are essentially different under loading and unloading conditions. At present, hydraulic fracturing technology for long horizontal wells is widely used in shale gas development [3]. Accurate understanding of strength and deformation characteristics of shale under unloading stress path is of great significance to the evaluation of borehole stability. Therefore, it is of great theoretical and engineering significance to study the mechanical properties of shale rock under different unloading rates and paths.

In recent years, the mechanical behavior of homogeneous rock during different unloading paths and rates has been widely investigated by means of experimental and theoretical analysis [4–10]. Conventional triaxial compression and unloading tests with different confining pressures and unloading rates were carried out on marble specimens [11]. The results show that the initial confining pressure and unloading rate have significant influence on the failure mode and strain energy conversion during unloading process. The effect of loading-unloading cycles on the mechanical behavior of mudstone was studied by ultrasonic velocity measurement. The results indicate that the physical and mechanical properties change associated with loading and unloading cycles. The mechanical deformation characteristics of sandstone under cyclic loading on uniaxial and triaxial compression were studied. The confining pressure has significant effect on deformation, the higher confining pressure results in larger strains [12].

The gas permeability tests of coal samples under different unloading paths were carried out to study the influence of unloading rate on the mechanical properties and permeability evolution. Research reports show that the higher the confining pressures unloading rate, the lower the compressive strength and plastic strain [13]. The permeability properties of coal under different unloading directions were carried out. The research obtains that when the unloading stress direction is perpendicular to bedding plane, more penetrating fractures and bedding fractures occur, and the permeability increases significantly [14]. Two different unloading tests were carried out on marble under different stress paths. The results show that compared with loading tests, the shear strength parameters and rupture modes are different under unloading confining pressure [15]. The mathematical and physical model of two-dimensional unloading mechanism of brittle rock under different stress paths is established by using general discrete element code PFC^2D^ numerical simulation method [16] The unloading confining pressure tests were carried out on marble, siltstone and coal, and it was found that rock burst under axial loading would absorb energy, while rock burst under unloading confining pressure would release energy [17].

There are few unloading tests for anisotropic rocks under different unloading stress paths. In general, the unloading confining pressure test is carried out with axial stress unchanged. Under different unloading methods and initial stress conditions, the mechanical properties of rock during unloading process are obviously different. In this study, the effects of unloading rate and stress path on the mechanical properties of gas-bearing shale are considered. The deformation and rupture characteristics of the same shale samples from a gas-bearing shale block are studied on MTS 815.04 rock mechanics test system, by means of unloading confining pressure tests with different unloading rates and initial confining pressure, and increasing axial stress-unloading confining pressure test. The test results are discussed, and the deformation, strength characteristics and failure behavior under different stress paths are studied. The unique mechanical properties of shale under different unloading stress paths are revealed, which can provide theoretical references for horizontal drilling, fracturing construction design and long-term fracture effectiveness analysis.

## 2. Experimental materials and procedure

### 2.1. Sample preparation

The material studied in this work is Longmaxi shale taken from Jiaoshiba site in Sichuan Basin,China. The shale is rich in organic matter and it can be seen a large number of graptolites, and deposited in the same period as the high-quality gas-bearing shale of the deep Longmaxi Formation. Compared with other shale, brittle mineral composition of gas-bearing shale is generally more than 50%, it is conducive to inducing fracture production and improving fracturing effect and it has obvious heterogeneity and anisotropy. Blocks of shale were collected from the exposed rock-mass section. The average mineralogical compositions are: 1.74% kaolinite, 1.42% plagioclasite, 3.15% illite, 58.70% quartz, 2.81% cristobalit, 12.64% albite, 5.59% calcite, 5.85% muscovite, 4.23% pyrite, 3.87% ankerite. The average density is 2.665 g/cm^3^ with 1.24% porosity. According to ISRM suggested methods, all the tests were performed on cylindrical samples with 25 mm in diameter and 50 mm in height, the surface parallelism value is within 0.03mm, and there was no obvious weak surface on the samples, all the samples were bored in the parallel directions to bedding planes. The P-wave velocities of the specimens ranged from 3600 to 4400 m/s, the uniaxial compressive strength is 124.26 MPa, and the tensile strength of vertical bedding surface is 10.23 MPa.

### 2.2 Experimental methods and test equipment

The loading and unloading tests were conducted on MTS 815.04 rock mechanics test system. The maximum axial load of the apparatus is 4600 kN, and the confining pressure can be up to 140 MPa. Both the axial and radial strains were recorded by strain gauge during the tests.

In this paper, in order to study the deformation and failure characteristics under unloading path and compare with conventional triaxial compression test, three groups of typical test schemes were designed. The detailed test conditions are listed as follows [18–19]:

Group I: Conventional triaxial compression test
  In order to design the stress level of unloading confining pressure and analyze the results of unloading tests, conventional triaxial compression tests were carried out. The design levels of confining pressures are 0, 20, 40 and 60 MPa. The test procedure shall be carried out according to the regulations. Displacement loading method was used in the test, and the loading rate is 0.12mm /min. The mechanical parameters of shale under conventional triaxial compression test are listed in Table 1.
Group II: Pre-peak constant maximum principal stress -unloading confining pressure test
  The experiment was divided into four steps: 1) Firstly, the hydrostatic pressure is gradually applied to the predetermined value; 2) Keeping the confining pressure constant, the deviatoric stress gradually increases to 80% of corresponding triaxial compressive strength. 3) The confining pressure is unloaded at a certain rate, while the axial stress is increased, that is, the maximum principal stress remains unchanged. 4) Instability ruptures stage.
Group III: Increasing axial stress and unloading confining pressure test
  The experiment was divided into four steps:1) Firstly, the hydrostatic pressure is gradually applied to the predetermined value; 2) Keeping the confining pressure constant, the deviatoric stress gradually increases to 80% of corresponding triaxial compressive strength., and the axial loading method was changed from displacement control to loading control at the unloading point;3) The confining pressure is unloaded at a certain rate, while the axial stress is increased, the increase rate of axial stress is greater than that of unloading confining pressure.4) Instability ruptures stage.

**Table 1:** The mechanical parameters of shale under conventional triaxial compression test.

Sample number	Confining pressure/MPa	Peak deviatoric stress /MPa	Elastic modus /GPa	Poisson’s Ratio
1–23	0	124.26	19.49	0.190
1–30	20	231.92	21.41	0.211
1–31	40	264.86	22.10	0.230
1–12	60	303.96	23.66	0.245

**Fig 1:**
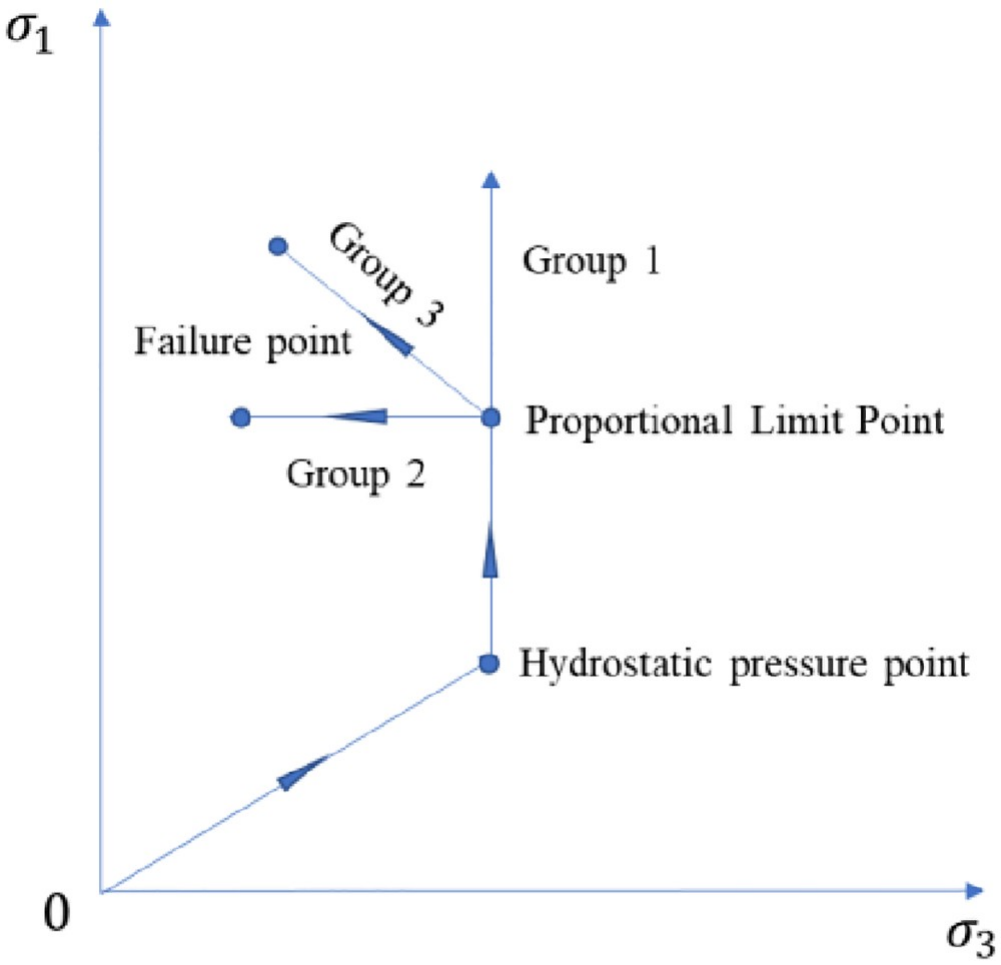
Stress path diagram of unloading test.

Stress path diagram of unloading test is shown in Fig 1. The test parameters of shale samples under unloading confining pressure test are listed in Table 2.

**Table 2:** The parameters of shale samples under unloading confining pressure test.

Sample number	Initial confining pressure/MPa	Initial stress ratio at unloading Point	Unloading condition
X-2	20	80%	0.4MPa/s UCP; 0.4MPa/s IAS
X-5	40	80%	0.4MPa/s UCP; 0.4MPa/s IAS
X-13	60	80%	0.4MPa/s UCP; 0.4MPa/s IAS
X-31	60	80%	0.6MPa/s UCP; 0.4MPa/s IAS
X-33	60	80%	0.8MPa/s UCP; 0.4MPa/s IAS
X-41	60	80%	1.0MPa/s UCP; 0.4MPa/s IAS
X-42	60	80%	0.4MPa/s UCP; 0.1MPa/s IAS
X-44	60	80%	0.6MPa/s UCP; 0.1MPa/s IAS
X-46	60	80%	0.8MPa/s UCP; 0.1MPa/s IAS

UCP = Unloading confining pressure; IAS = Increasing axial stress.

## 3. Experimental results and analysis

### 3.1 Analysis of deformation characteristics

**Fig 2:**
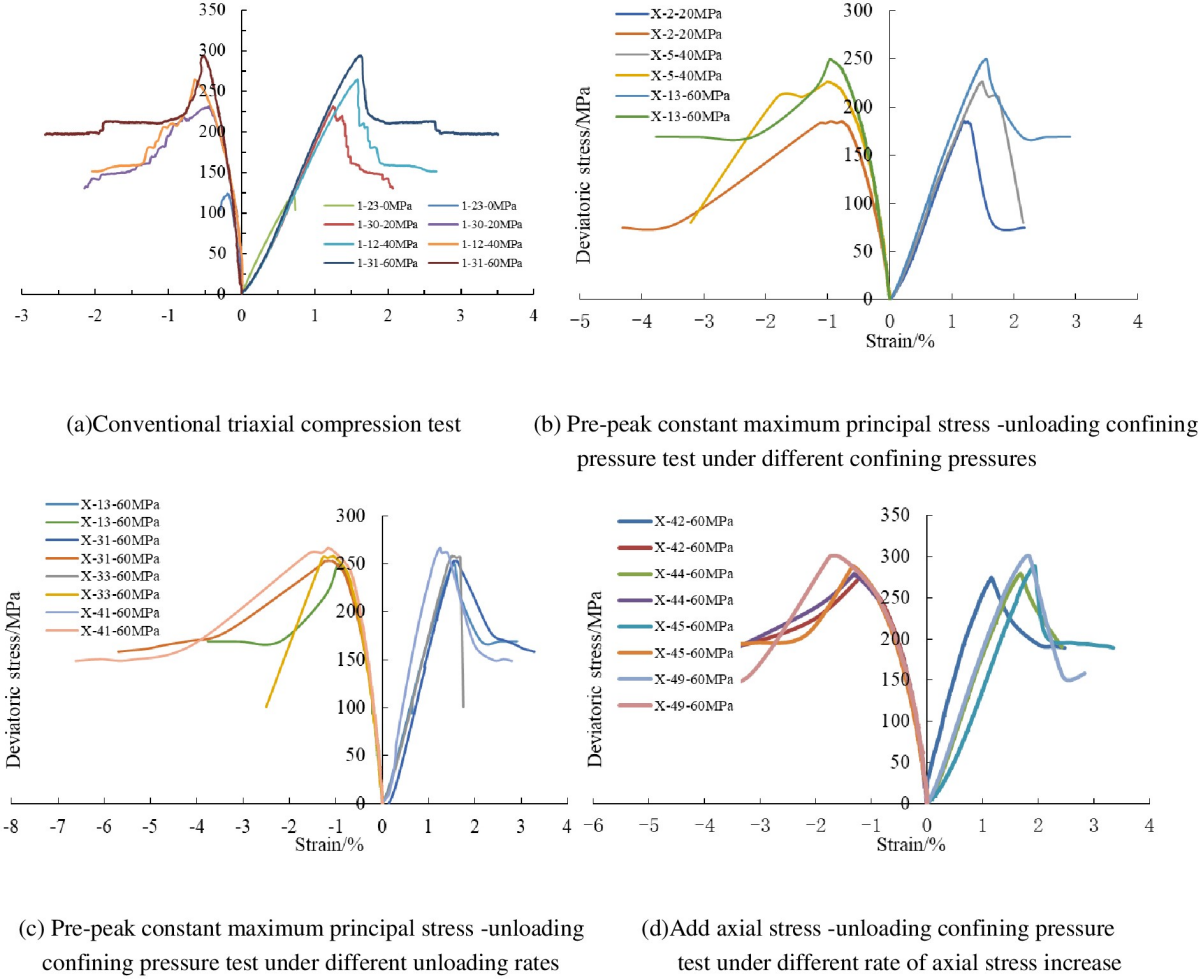
Typical complete stress-strain curves of shale rock under different stress paths.

Typical complete stress-strain curves of shale samples under different stress paths were shown in Fig 2. As can be seen from Fig 2(a), conventional triaxial compression tests show obvious elastic-plastic characteristics, the samples still have a certain bearing capacity after reaching its peak strength, and the residual strength increases with the increasing of confining pressure, while unloading confining pressure tests show elastic-brittle characteristics, when the samples reach the peak strength, it is damaged severely and loses its bearing capacity completely, as shown in Fig 2(b), 2(c) and 2(d). With the increasing of confining pressure, the peak axial strain increases gradually, the stress-strain curve shows a nearly linear relationship, after reaching the peak strength, the deviatoric stress decreases obviously, especially under unloading confining pressure, the deviatoric stress drops rapidly after reaching peak strength, and brittle failure occurred in the sample.

Under different stress paths, the axial strain increases with the increasing of initial unloading confining pressure. The main reason is that with the increase of initial confining pressure, the axial bearing capacity is strengthened and its axial ultimate strain is increased. Under unloading condition, when the sample ruptured, the corresponding axial strain is greater than the radial strain. The failure strain of shale samples under unloading confining pressure test were shown in Table 3.

**Table 3:** Failure strain of shale samples under unloading confining pressure tests.

Sample number	Confining pressure at rupture/MPa	Peak deviatoric stress/MPa	Axial strain at fracture/%	Radial strain at fracture/%
X-2	14.11	185.21	1.26	-0.97
X-5	32.73	226.33	1.49	-1.01
X-13	50.20	249.94	1.56	-0.96
X-31	45.82	253.01	1.62	-1.25
X-33	41.42	258.48	1.51	-1.06
X-41	34.91	267.10	1.24	-1.17
X-42	51.71	274.66	1.16	-1.20
X-44	53.70	279.65	1.68	-1.31
X-45	54.74	288.60	1.94	-1.33
X-49	54.15	301.36	1.81	-1.63

**Fig 3:**
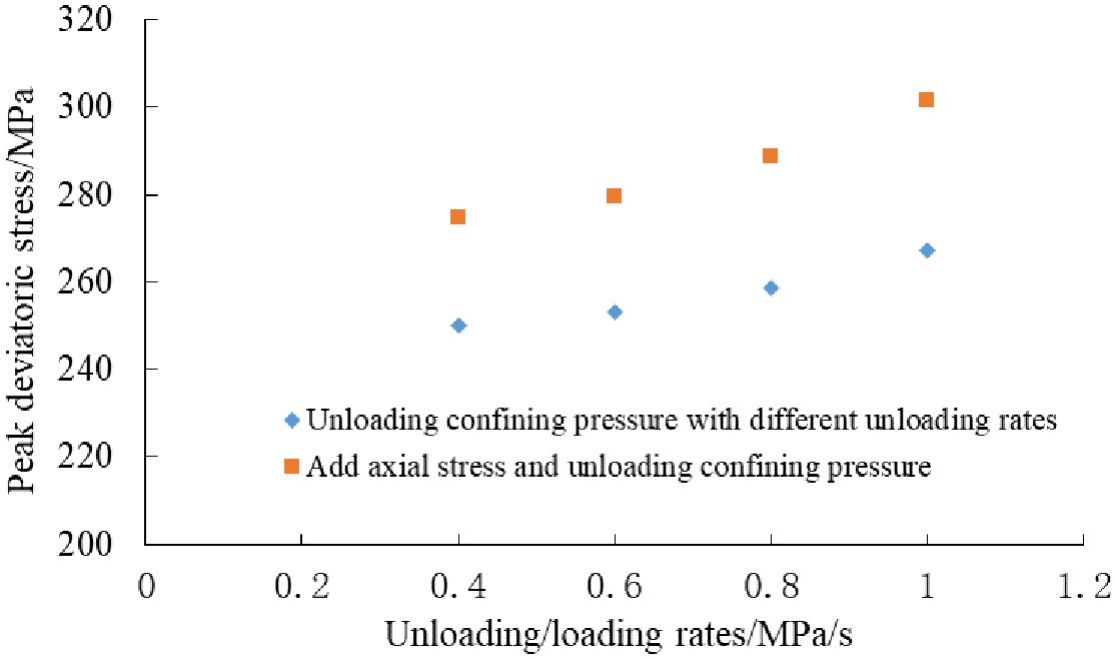
The relationship between peak deviatoric stress and different unloading rates.

The relationship between peak deviatoric stress and unloading rates is shown in Fig 3. It can be obtained from Table 3 and Fig 3, under the same initial confining pressure (60MPa), the higher the unloading rate, the higher the peak deviatoric stress is. This indicates that under high unloading rate, the internal micro-cracks induced by unloading lag behind the stress response, and the peak deviatoric stress increases relatively. However, after unloading test, the samples were broken more fully. During unloading process, the energy of micro cracks accumulates, which leads to a more complex fracture morphology. Under the condition of add axial stress (The rate is 0.4, 0.6, 0.8, 1.0MPa/s) and unloading confining pressure (The rate is 0.1MPa/s), the peak deviatoric stress increases with the increasing of axial stress rate. It also shows that with the increasing of loading rate, the strain lags behind the stress. The unloaded sample produced multiple fracture surfaces.

The failure in loading test is caused by compression (mainly axial) deformation, while the failure in unloading test is caused by the strong expansion in the unloading direction, even when unloading occurred in both directions, strong expansion can lead to destruction of shale samples. The failure strain under different initial unloading confining pressures shows that: under the same stress path, the higher the initial confining pressure is, the more serious failure is.

### 3.2 Analysis of unloading strain characteristics

**Fig 4:**
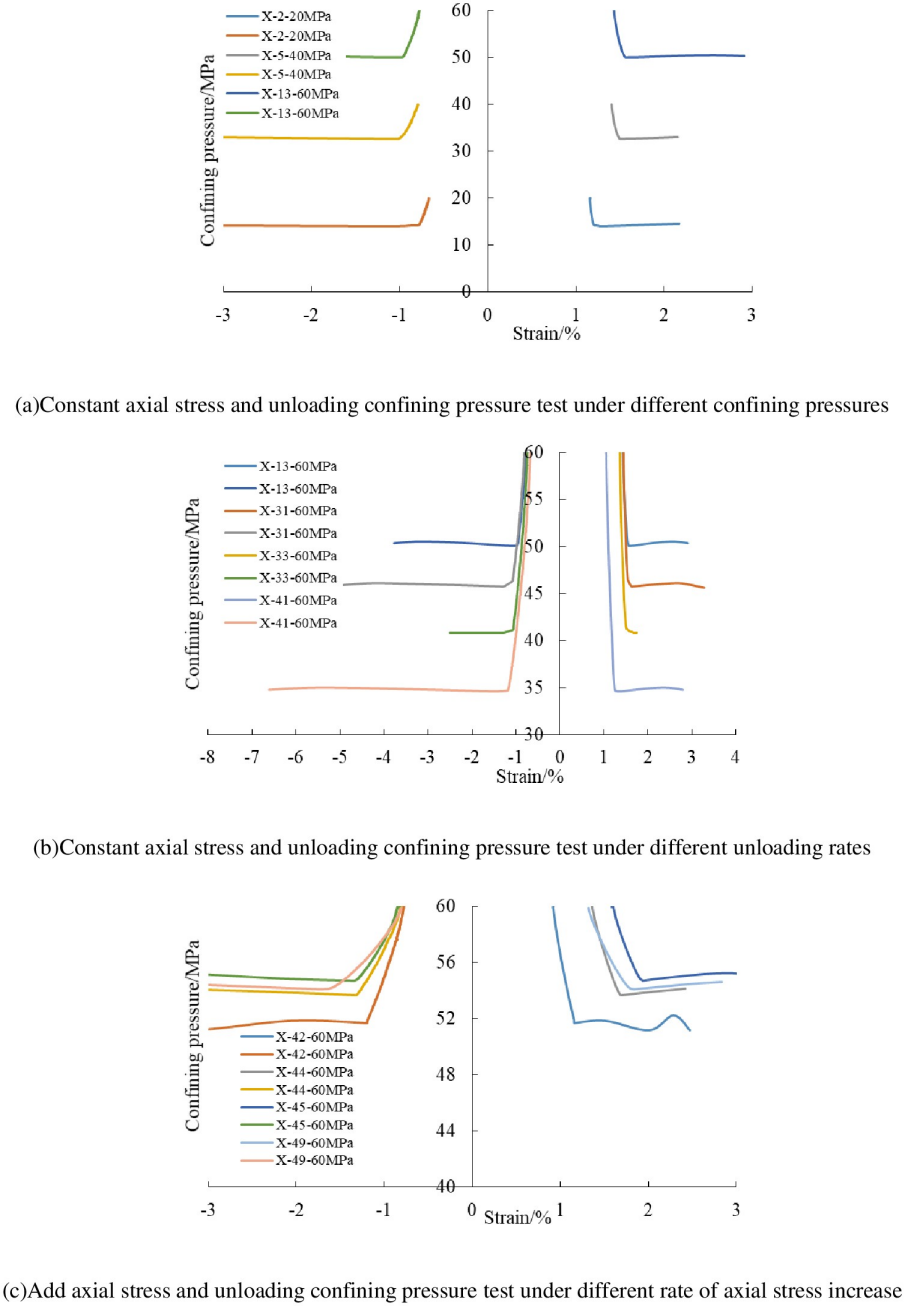
The relationship between confining pressure and strains of shale specimens under unloading tests.

The relationship between confining pressure and unloading strain under unloading test is shown in Fig 4. In the initial stage, the deformation under two different unloading paths increases slowly. With the decreasing of confining pressure, the samples were in elastic stage. However, with the confining pressure continues to unloading, the axial and radial strain of samples increase sharply. There is an obvious nonlinear relation with the confining pressure, and the sample appeared radial expansion deformation. Before the sample is unloaded to failure, the increase of axial strain is small. When the sample is unloaded to a certain critical, the axial strain begins to increase and until the sample destroyed, indicating that the unloading failure are mainly manifested as radial deformation.

As shown in Table 4, when the initial confining pressure is 20 MPa, the axial strain variation caused by unloading is only about 31% of the radial strain variation before the sample is unloaded to failure. Under high confining pressure (60 MPa); the axial strain variation caused by unloading is about 54% of the radial strain variation. On the whole, at the same initial unloading confining pressure, the ratio decreases with the increasing of unloading rate. When the unloading rate increases, the radial deformation is large and the sample shows obvious volume expansion. This indicates that the brittle fracture characteristic under unloading condition increases with the increasing of unloading rate and initial confining pressure.

**Table 4:** The ratio of the change of axial strain to radial strain in unloading tests.

Sample number	Axial strain at initial point of unloading/%	Axial strain at unloading termination point/%	Radial strain at initial point of unloading/%	Radial strain at unloading termination point/%	Ratio of axial to radial strain increment
X-2	1.154	1.305	-0.666	-1.148	0.31
X-5	1.395	1.591	-0.785	-1.406	0.32
X-13	1.432	1.691	-0.777	-1.258	0.54
X-31	1.442	1.641	-0.803	-1.291	0.41
X-33	1.359	1.519	-0.737	-1.058	0.50
X-41	1.054	1.287	-0.673	-1.321	0.36
X-42	0.916	1.158	-0.777	-1.199	0.57
X-44	1.364	1.696	-0.816	-1.322	0.66
X-45	1.584	1.938	-0.833	-1.340	0.70
X-49	1.317	1.847	-0.805	-1.752	0.56

Under the condition of add axial stress and unloading confining pressure, axial loading actually intensifies the axial and radial deformation in rock mass, the increase of axial stress causes the increase of axial strain, the ratio of the change of axial strain to radial strain is relatively large.

### 3.3 Analysis of stress path and strain

**Fig 5:**
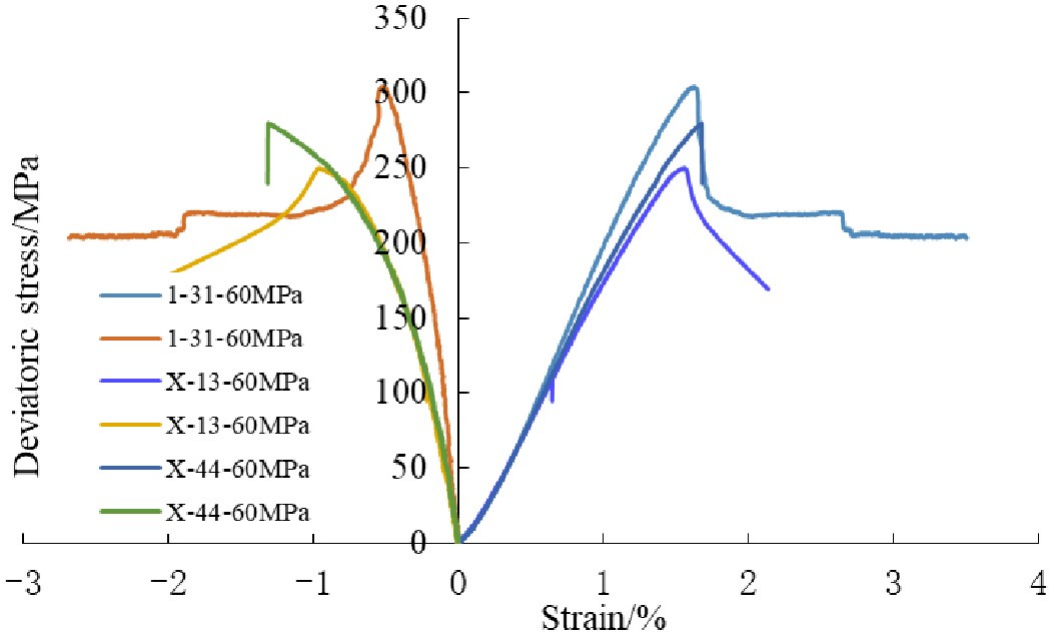
Stress-strain curves of shale samples under different stress paths.

The influence of stress paths on deformation characteristics is analyzed by comparing with conventional triaxial compression tests. Fig 5 shows the stress-strain curves of shale samples under confining pressures of 60MPa; including constant axial stress-unloading confining pressure tests, add axial stress-unloading confining pressure tests and conventional triaxial compression tests.

As can be seen from Fig 5, the stress-strain curves coincide with each other and show a nearly linear relationship before reaching peak stress. Then, with the axial stress increasing, the stress-strain curves begin to deviate: Compared with conventional triaxial compression test and unloading confining pressure test. The axial stress during unloading failure is smaller than that of conventional triaxial compression test, which indicates that unloading path accelerates the failure and reduces the bearing capacity.

Under the same initial confining pressure and different stress paths, radial strain in failure: Add axial stress-unloading confining pressure test > Pre-peak constant maximum principal stress -unloading confining pressure test > conventional triaxial compression test. The test results show that under the same initial stress, the brittle characteristics in unloading confining pressure are more obvious and the expansion is more intense. This is because under the same initial confining pressure, the failure intensity of axial stress-unloading is significantly greater than that of loading. In addition, the samples in add axial stress-unloading test accumulated more energy before failure. During the process of unloading, the energy is still absorbed from outside, which leads to the violent expansion of rock mass, and the stress drop is obvious.

### 3.4 Strength parameter characteristic

**Fig 6:**
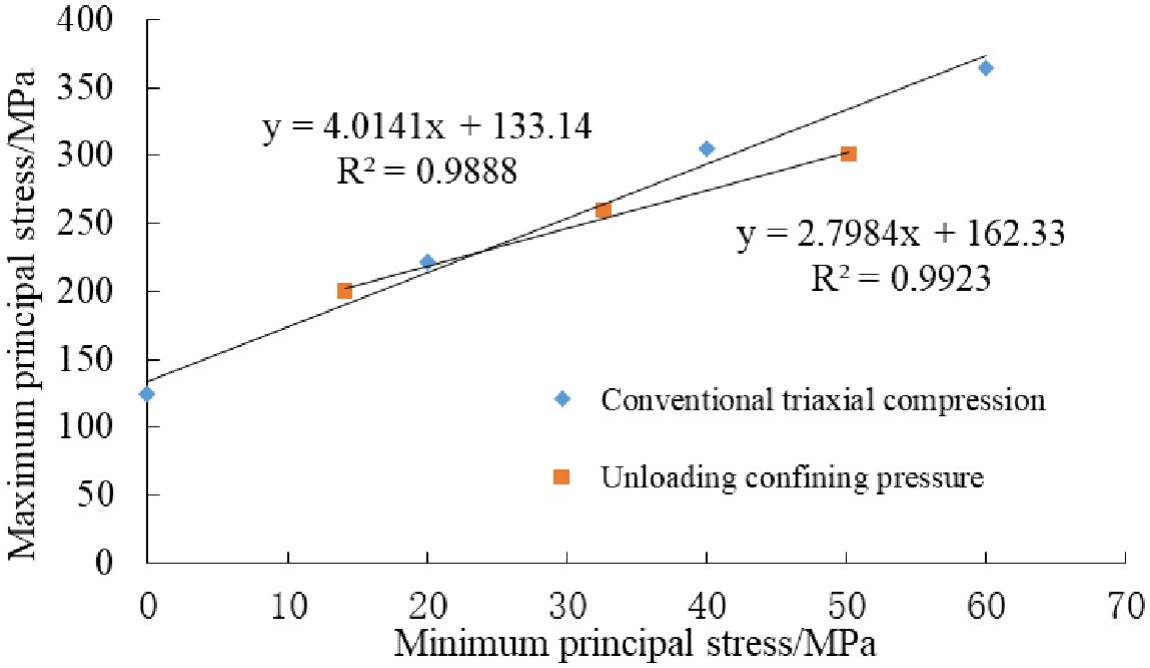
The relationships between maximum and minimum principal stress.

In Mohr-Coulomb yield criterion, there is a linear relationship between the maximum principal stress and minimum principal stress. The relationship between peak stress and confining pressure under different stress paths was statistically analyzed, and the results were shown in Fig 6. The peak stress is sensitive to confining pressure. With the increasing of initial confining pressure, the peak stress increases linearly. Previous studies by scholars have shown that the strength of rock is not only related to the geological environment, but also to the stress path of loading and unloading [20–21].

Mohr-coulomb (M-C) strength criterion is one of the most widely used strength criterion in geotechnical engineering. The expression of M-C strength criterion is:

τ=c+σtanφ (1)

In the formula:*τ* and *σ* are the shear stress and normal stress on the shear failure surface, respectively. For triaxial compression test, *σ* and *τ* can be expressed as:

$$\begin{array}{l} \sigma =\frac{1}{2}(\sigma_{1}+\sigma_{3})+\frac{1}{2}(\sigma_{1}-\sigma_{3})\;\text{cos}\;(2\varphi )\\ \tau =\frac{1}{2}(\sigma_{1}-\sigma_{3})\;\text{sin}\;(2\varphi ) \end{array}\}$$ (2)

Using the results of triaxial compression test, the following linear relations can be obtained:

$$\sigma_{\text{1}}=m\sigma_{3}+b$$ (3)

In the formula: *m* and *b* are the slope and intercept of the fitted line respectively. According to the parameters *m* and *b*, the cohesive force and internal friction angle can be determined.

The shear strength parameters of shale rock under different stress paths calculated by formula (1) ~ (3) are shown in Table 5. The relationships between maximum and minimum principal stress is shown in Fig 6.

**Table 5:** Shear strength parameters of shale sample under different stress paths.

Test type	*m*	*b*	*R*^2^	*c*/MPa	*φ*/°
Conventional triaxial compression	4.0141	133.14	0.9888	33.23	36.95
Unloading confining pressure	2.7984	162.33	0.9923	48.52	28.26

As can be seen in Table 5, the shear strength parameters were obtained by unloading and loading are different. Previous studies by scholars have found that [17–18], compared with conventional triaxial compression test, the cohesion value decreased and the internal friction angle increased in unloading confining pressure test for marble. However, the law of shale obtained in this study is just the opposite. The cohesion value obtained from unloading test is increased and the internal friction angle decreased. These indicate that the regularity gained from the use of homogeneous rocks is no longer applicable in stratified shale. The existing stratification plays an important role in its failure. This is consistent with the fact that after damaged, multiple fracture surfaces were generated and some bedding surfaces were opened.

### 3.5 Failure characteristic analysis

**Fig 7:**
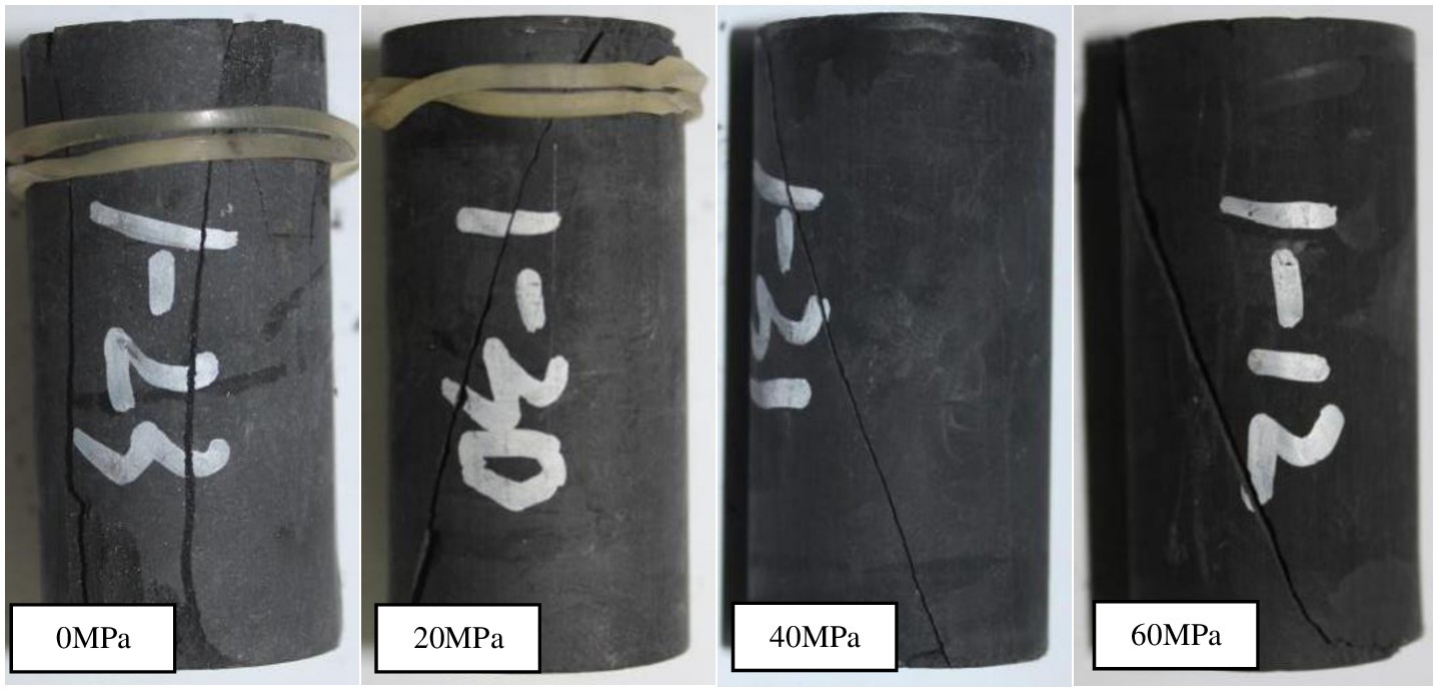
Fractured samples after conventional triaxial compression test.

Typical fractured shale samples after conventional triaxial compression test are shown in Fig 7. Under conventional triaxial compression, there is a clear sound when the sample broken, it shows typical brittle failure characteristics. The macroscopic failure modes are mainly split and shear failure. The splitting failure occurs when the confining pressure is 0 MPa (UCS). The sample was splitted into several vertical cracks along bedding plane. The angle between the splitted failure surface and maximum principal stress is small and approximately parallel.

Moreover, shear failure occurs under confining pressure and is mainly manifested as single shear failure [22–23]. With the increase of confining pressure, the roughness of the shear fracture surface decreases. The main shear plane runs through the whole sample and is cut into two triangular vertebrae, and there are a large number of scratches on the failure surface. Some of which even appear as powder, which is caused by secondary shear failure caused when the damaged triangular vertebral rock blocks resist the loading during shear slip process. When the sample is destroyed, it appears a micro-drum shape, resulting in volume expansion.

**Fig 8:**
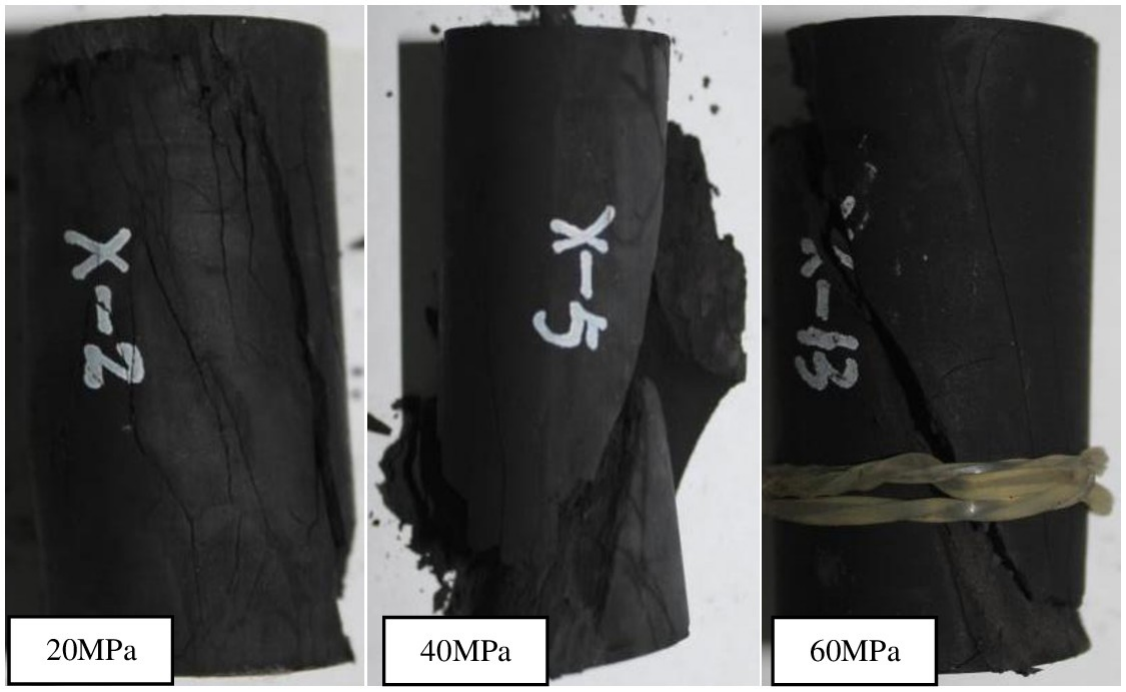
Fractured samples after unloading with different initial confining pressures.

The fractured shale samples after unloading with different initial confining pressures are shown in Fig 8. Unloading deformation is characterized by strong expansion or expansion along unloading direction. Compared with conventional triaxial compression test, unloading is easier to deformation and rupture, and crack damage and plastic deformation are smaller. Under low initial confining pressure, multiple oblique shear fracture surfaces were generated. The damaged sample has strong tensile fracture characteristics, including shear and tensile fracture modes. There are several micro-tensile fracture planes sandwiched between shear fracture planes. This indicates that there is a joint action of tension and shear failure. However, with the increasing of unloading confining pressure, the proportion of shear failure components increases. The failure mode is from tensile-shear failure to shear failure. The shear fracture angle increases with the increasing of rupture confining pressure. Compared with conventional compression test, the fracture characteristics of unloading samples are much more complicated.

**Fig 9:**
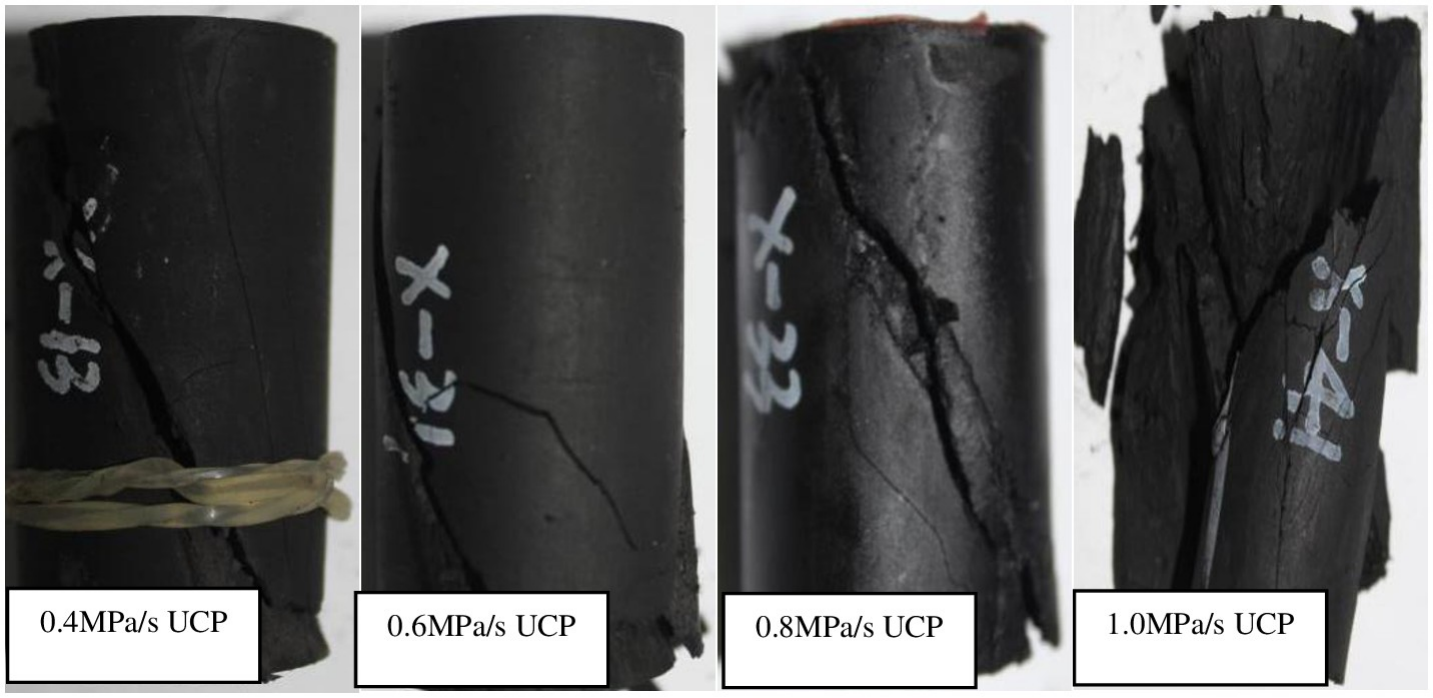
Fractured samples after unloading with different unloading rate.

The fractured samples after unloading with different unloading rate are shown in Fig 9. It can be seen that when the rate of unloading confining pressure increases, the fracture degree becomes more complex. The fracture surface penetration and the tensile crack morphology increase. Multiple secondary fracture surfaces were created around the main fracture. When the rate of unloading is 1.0MPa /s, there are even tensile cracks nearly perpendicular to the unloading direction.

**Fig 10:**
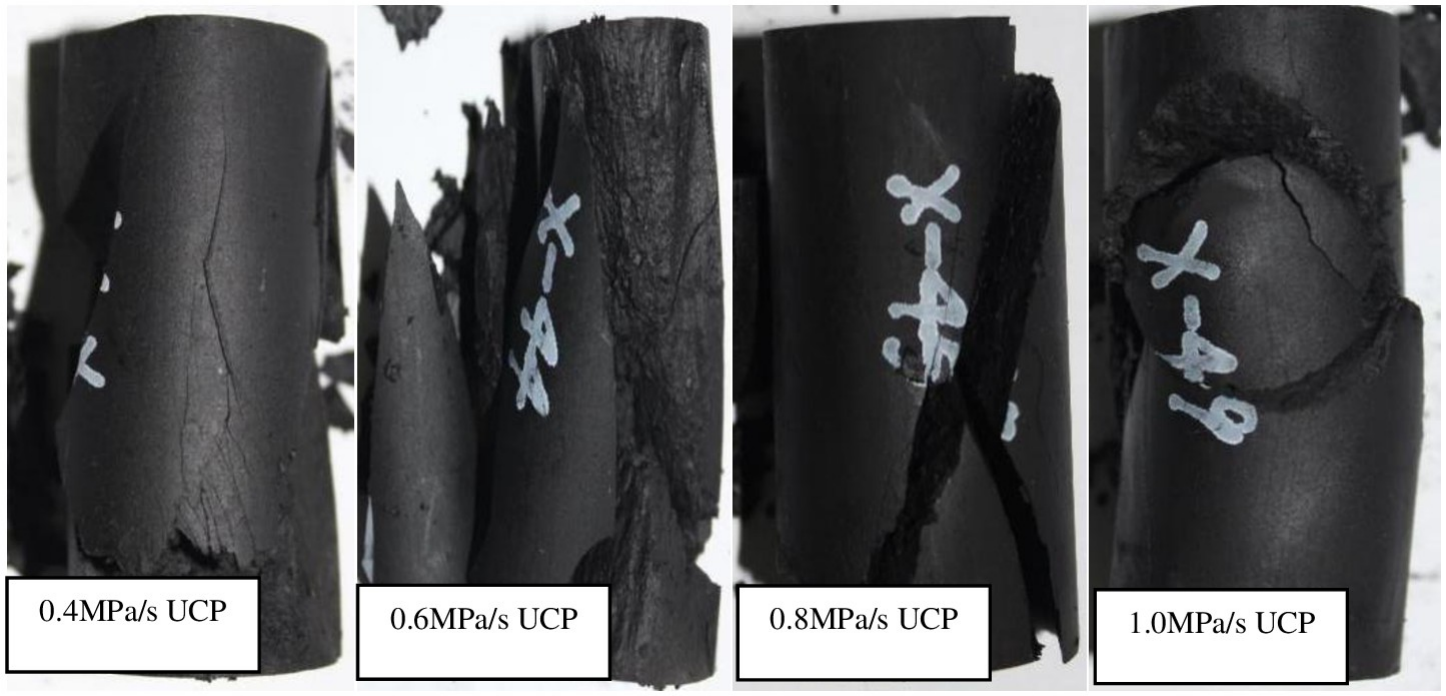
Fractured samples after add axial stress -unloading confining pressure test with axial stress increases under different rates.

The fractured samples after add axial stress -unloading confining pressure test with axial stress increases under different rates are shown in Fig 10. Under the conditions of increasing axial stress-unloading confining pressure, various types of tensile and shear fractures with different mechanisms are developed. The fracture pattern is more complicated than the two schemes. The main fracture of sample X-45° is conjugate X or local shear failure. Since the sample is under axial loading all the time, during unloading confining pressure, the sample absorbs the work done by axial stress and converts it into corresponding strain energy and surface energy. Therefore, axial stress also plays a role in promoting the damage and fracture during unloading, accelerating the process of crack expansion and strengthening the scale of crack expansion.

There are great differences in fracture characteristics of samples under different loading and unloading stress paths. It can be seen from the failure morphology in Figs 7 ~ 10. The fracture morphology after conventional triaxial compression is dominated by single shear fracture. The fracture surface morphology under unloading stress path is more complex and mainly composed of tension and shear. Therefore, during the process of drilling long horizontal well, the horizontal well is more prone to tensile and shear damage, it leads to hole wall instability. More attention should be paid to the mechanical behavior of gas-bearing shale under unloading conditions.

## 4. Conclusions

In this paper, conventional triaxial compression, unloading tests under different initial confining pressures and unloading rates were conducted. The main conclusions are as follows:

1. Under different stress paths, the axial strain increases with the increasing of initial unloading confining pressure, in the unloading stage, the change of axial strain is less than radial strain, and the samples are mainly destroyed by dilation instability.
2. Under the same stress path, the higher the initial confining pressure is, the more severe the sample failure is. The brittle rupture characteristic under unloading condition increases with the increasing of unloading rate and initial confining pressure.
3. At the same initial confining pressure, under high unloading rate, the internal micro-cracks induced by unloading lag behind stress response, and the peak stress increases relatively.
4. Compared with conventional triaxial compression test, unloading confining pressure is more prone to deformation and fracture, and the degree of failure is more intense. When the rate of unloading confining pressure increases, the damage is more complicated.

## Supporting information

S1 Dataset Data availability files. (XLS)
